# Synthetic biology, genetic circuits and machine learning: a new age of cancer therapy

**DOI:** 10.1002/1878-0261.13420

**Published:** 2023-04-01

**Authors:** Krishneel Prasad, Ryan S. Cross, Misty R. Jenkins

**Affiliations:** ^1^ Immunology Division The Walter and Eliza Hall Institute of Medical Research Parkville Vic. Australia; ^2^ The Department of Medical Biology The University of Melbourne Parkville Vic. Australia; ^3^ Department of Biochemistry and Chemistry, Institute for Molecular Science La Trobe University Bundoora Vic. Australia

**Keywords:** chimeric antigen receptor T cells, gene circuits, immunotherapy, signalling, synthetic biology

## Abstract

Synthetic biology has made it possible to rewire natural cellular responses to treat disease, notably demonstrated by chimeric antigen receptor (CAR) T cells as cancer immunotherapy. Building on the success of T‐cell activation using synthetic receptors, the field is now investigating how induction of noncanonical signalling pathways and sophisticated synthetic gene circuitry can enhance the antitumour phenotype of engineered T cells. This commentary explores two recently published studies that provide proof of concept for how new technologies achieve this. The first demonstrated that non‐naturally occurring combinations of signalling motifs derived from various immune receptors and arranged as a CAR drove unique signal transduction pathways in T cells and improved their tumour killing ability. Here, machine learning complemented the screening process and successfully predicted CAR T‐cell phenotype dependent on signalling motif choice. The second explored how synthetic zinc fingers can be engineered into controllable transcriptional regulators, where their activity was dependent on the presence or absence of FDA‐approved small‐molecule drugs. These studies are pivotal in expanding the design choices available for gene circuits of the future and highlight how a single cellular therapy could respond to multiple environmental cues including target cell antigen expression, the tumour microenvironment composition and small molecule drugs.

## Graphical abstract

This commentary explores how the merger between artificial intelligence and synthetic biology has recently enabled the development of novel, engineered T cells for cancer immunotherapy. We discuss two recent studies that push the barriers of synthetic receptor and gene circuit design by presenting new library screens and genetic tools that allow tuning and regulation of the signalling pathways recruited following T‐cell activation.

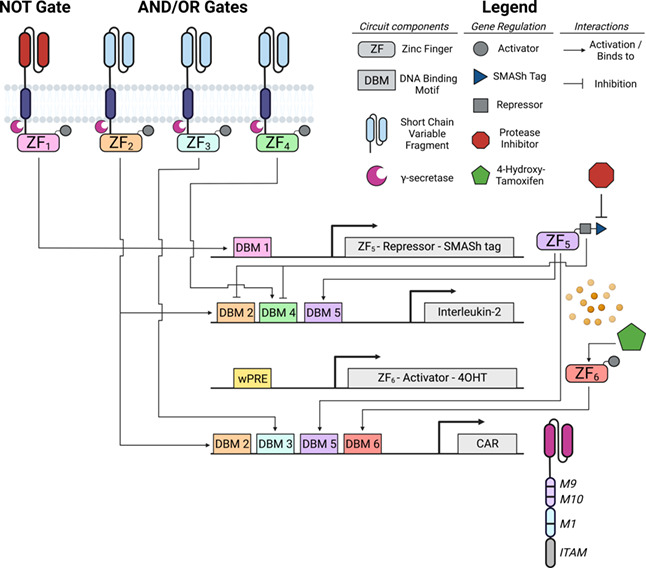

**Abbreviation**
CAR: chimeric antigen receptor

Cells communicate with each other and coordinate their functions through the exchange of various molecular signals. As we gain a deeper understanding of the role of individual signalling molecules and sequence motifs, the synthetic biology field applies engineering principles to redesign these natural cellular processes. While there are concerns about the potential risks of synthetic biology, it has the potential to revolutionise the development of cancer therapy. This can be achieved by creating synthetic molecules and modifying cells to perform functions beyond their natural capability. Thus far, the use of monoclonal antibodies has allowed biologists and oncologists to manipulate native biology, but this approach is naturally limited. Synthetic biology represents a new and innovative way of generating ‘user‐controlled’ genetic circuits to overcome limitations imposed by nature.

In the realm of immune cell programming, synthetic biology has significantly impacted the development of chimeric antigen receptor (CAR) T cells [1]. These CARs offer a modular design that can be tailored to enhance specificity, safety and functionality. By combining the strength of CAR T cell biology with the manipulation of gene transcription using genetic circuits, the field is moving from empirical protein engineering to specified design driven by a deeper understanding of how each modular component impacts cellular biology.

Recently, two remarkable articles were published in *Science*, showcasing the practical application of synthetic biology to cutting‐edge therapies. Daniels et al. [2] engineered a library of ~ 1200 receptors (new sentences) using 12 diverse ‘atomic’ signalling motifs (words) and used machine learning and neural network analysis to assess their functional consequences in CAR T cells (language). The study revealed a set of rules for CAR signalling that led to new phenotypes not previously seen with traditional receptor design in the native state. By incorporating an SH2 motif to recruit PLC*γ*1, the team was able to improve the CAR costimulatory function of 41BB‐like, but not CD28‐like, CAR signalling as measured by cytotoxicity (shown by CD107a^+^ exposure as a surrogate for degranulation) and memory potential (IL7a^+^/KLRG1^−^) [2]. Whilst CD28 and 41BB costimulatory domains are characterised in CAR T cells [3], the authors show that synthetic motifs facilitate the activation of noncanonical signalling pathways, enabling a customised, novel T cell function [2]. This study demonstrates the potential of machine learning and neural networks in understanding the relationship between motif choice, combination and position to tailor synthetic receptors for desired T‐cell phenotypes [2].

Similarly, Li et al. [4] made significant advancements and established proof of concept for synthetic transcription factors for clinical use. Zinc fingers are the most prevalent DNA‐binding domains in human transcription factors, comprising around 30 amino acids that each recognise ~ 3 base pairs of DNA. Adapting Cys2His2 zinc fingers, Li et al. established proof of concept of synthetic transcription factors, termed synZiFTR, capable of recognising an 18 bp unique artificial minimal promoter, absent from the human genome. Li et al. then generated 11 synZiFTRs to significantly advance previously available transcriptional activators such as Gal4, TetR, tTA or ZFHD1 and demonstrated that these synZiFTRs can create synthetic circuits running parallel transcription in human cells. The authors further demonstrated the capacity for molecular control of synZiFTR activity through three FDA‐approved drugs *in vitro*. Using the antiviral grazoprevir to induce an anti‐HER2 CAR in T cells specifically and tamoxifen (4OHT/TMX) to induce ‘super’ high‐affinity IL‐2 (serving as a titratable proliferation gene switch), they demonstrate inducible antitumour efficacy in NALM6 tumour‐bearing mice [4]. These molecular switches facilitated individual, sequential or concordant expression to refine and enhance therapeutic ability.

Although other inducible synthetic circuits could mitigate risks associated with CAR T cell therapy [5, 6], the inducible synZiFTR system unlocks the potential for gene circuit design of greater complexity by incorporating antigen recognition, autoregulation and drug‐inducible switches into the circuitry (Fig. 1). Gene circuitry arising from the merger between artificial intelligence and synthetic biology will therefore contain a greater number of genes compared with immunotherapies currently being tested. Accordingly, advances in nonviral gene transfer systems will in the future facilitate the expression of larger synthetic genes in primary human cells [7]. This has the potential to revolutionise immunotherapy by providing modular synthetic gene design choices based on desired phenotypic outputs. For instance, whilst CD8^+^ T cell proliferation and function can be sustained for many years using vaccine boosters, [8] improving CAR T cell longevity and reducing exhaustion may require genetic circuits that emulate the stimulation protocols described in this study.

**Fig. 1:**
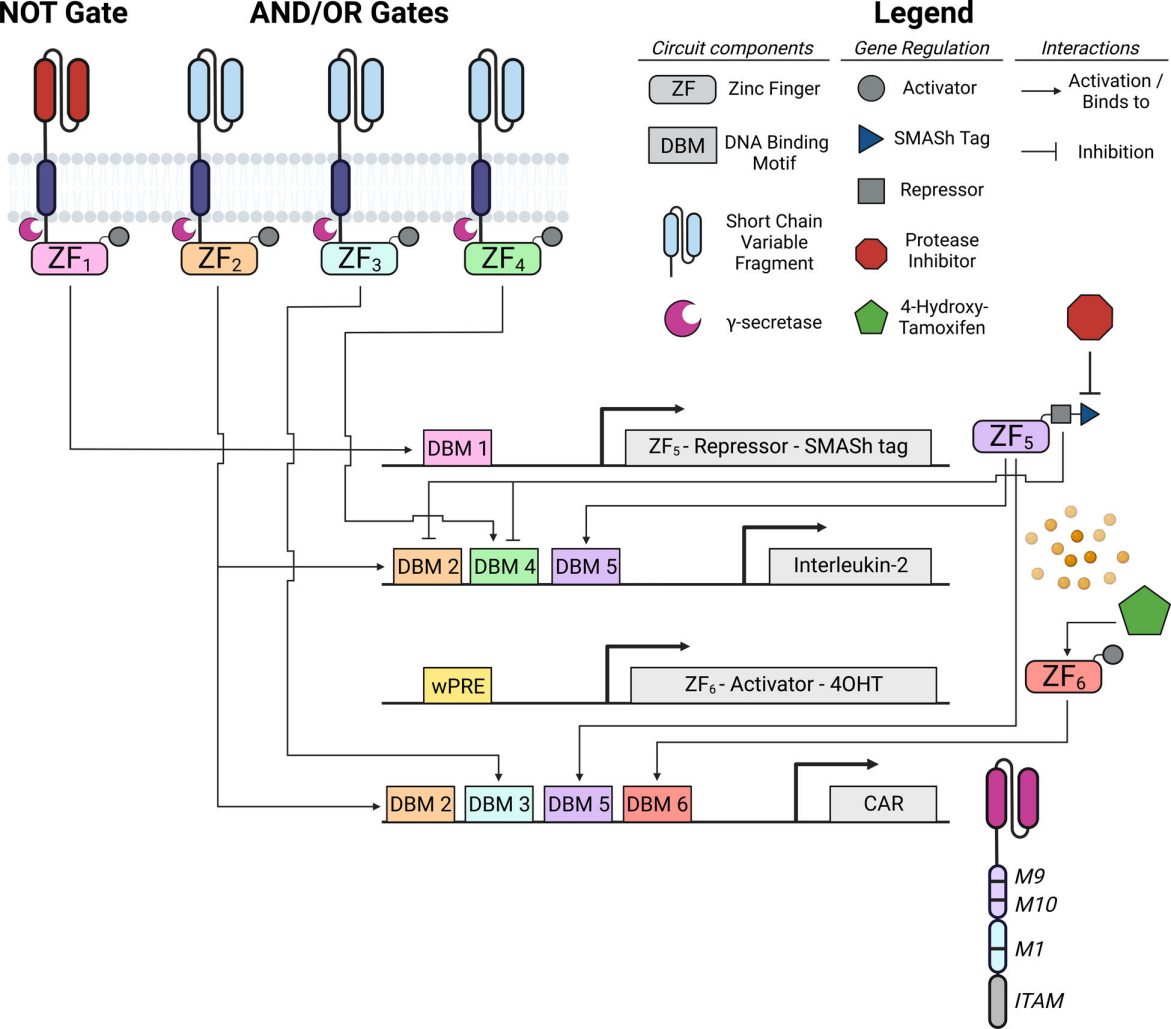
A theoretical gene circuit incorporating multiple synthetic design elements to tightly regulate transgene expression. Antigen recognition via ‘SynNotch‐like’ γ‐secretase cleavage receptors liberate synthetic zinc finger transcriptional regulators to drive distinct downstream signalling cascades leading to gene activation (AND/OR gates) or repression (NOT gate). Inclusion of multiple DNA‐binding motifs (DBM) creates synthetic promoters adding layers of transcriptional regulation based on cell stimulus. Inclusion of drug binding sites on synthetic proteins or protein tags (e.g. SMASh) allows for user‐override of the gene circuit via delivery of small molecule drugs (e.g. 4‐OHT) to immediately achieve a desired outcome (e.g. CAR expression or removal of repressive elements). 4‐OHT, 4‐hydroxyl‐tamoxifen; CAR, chimeric antigen receptor; SMASh, small molecule assisted shutoff. Created with BioRender.com.

Both studies present the exciting outcome that synthetic signalling components can create T cell phenotypes with superior efficacy, different from endogenous T cells that only signal through canonical activation pathways. The complexity of synthetic receptor library screens will continue to increase as multiple parameters are tuned simultaneously (e.g. Costimulatory domain choice, position, number, proximity to the membrane and receptor affinity). These screens consider synthetic receptors to be built from modular functional units and have extended from improving CAR design to now being used to create clinically translatable gene circuits regulated by synthetic intramembrane proteolysis receptors [9]. Researchers must keep in mind that the quality and depth of experimentally validated data used for machine learning training can limit its predictions. As training data improve, more nuanced cellular programs will be predicted by artificial intelligence, thereby accelerating the rate at which improved immunotherapies can be preclinically characterised. Although Daniels et al. and Li et al. validate these new technologies in the context of cancer immunotherapy, their versatility may be essential in how we view and treat other diseases in the future, such as autoimmunity and infection.

## Conflict of interest

The authors declare no conflict of interest.
